# Vacuum-assisted breast biopsy vs core needle biopsy: a systematic review and meta-analysis

**DOI:** 10.1007/s00330-025-12299-1

**Published:** 2026-01-19

**Authors:** Nisha Sharma, Sina Theis, Tobias Vogelmann, Ruud Pijnappel

**Affiliations:** 1 Leeds Teaching Hospitals NHS Trust, Leeds, UK; 2 LinkCare GmbH, Ludwigsburg, Germany; 3 Department of Radiology at the University Medical Centre Utrecht, Utrecht, The Netherlands

**Keywords:** Biopsy, Systematic review, Biopsy, Large-core needle, Breast neoplasms

## Abstract

**Objectives:**

Vacuum-assisted breast biopsy (VABB) and core needle biopsy (CNB) are percutaneous biopsy methods used for the assessment of suspicious breast lesions. This systematic review and meta-analysis focused on comparative diagnostic performance outcomes of lesions biopsied with VABB or CNB.

**Materials and methods:**

Studies comparing VABB to CNB were searched in PubMed and Cochrane Library. Pooled risk ratios (RR) with 95% CI using random-effects models were calculated for atypical ductal hyperplasia (ADH) and ductal carcinoma in situ (DCIS) underestimation rates, repeat biopsy rate, concordance rate, calcification retrieval rate, and false-negative rate. Sensitivity analyses were performed using the leave-one-out approach. Risk of bias (RoB) was assessed using the quality assessment of diagnostic accuracy studies (QUADAS)-2 tool.

**Results:**

Sixty studies were included from 937 records identified. ADH (RR: 0.63, 95% CI: 0.55–0.72, 22 studies) and DCIS (0.47, 0.39–0.58, 27 studies) underestimation was significantly lower with VABB compared to CNB. The repeat biopsy rate was significantly lower with VABB than with CNB (0.78, 0.69–0.88, 9 studies). VABB increased the likelihood that the surgical histology will match the biopsy (1.07, 1.04–1.11, 12 studies). The calcification retrieval rate was estimated to be significantly higher when using VABB (1.09, 1.04–1.14, 11 studies). Two-thirds of all studies had a low RoB.

**Conclusion:**

VABB, as a first-line diagnostic procedure, is superior to CNB in terms of delivering a definitive diagnosis and reducing upgrade rates to malignancy, delivering a safe and efficient patient workflow.

**Key Points:**

***Question*** *What is the diagnostic performance of VABB vs CNB in assessing suspicious breast lesions, including those with and without calcifications?*

***Findings****Meta-analysis results showed a significantly lower risk for DCIS underestimation, ADH underestimation, and repeat biopsies using any imaging-guided VABB compared to imaging-guided CNB*.

***Clinical relevance****VABB, as a first-line diagnostic procedure, is superior to CNB in terms of delivering a definitive diagnosis and reducing upgrade rates to malignancy, delivering a safe and efficient patient workflow*.

**Graphical Abstract:**

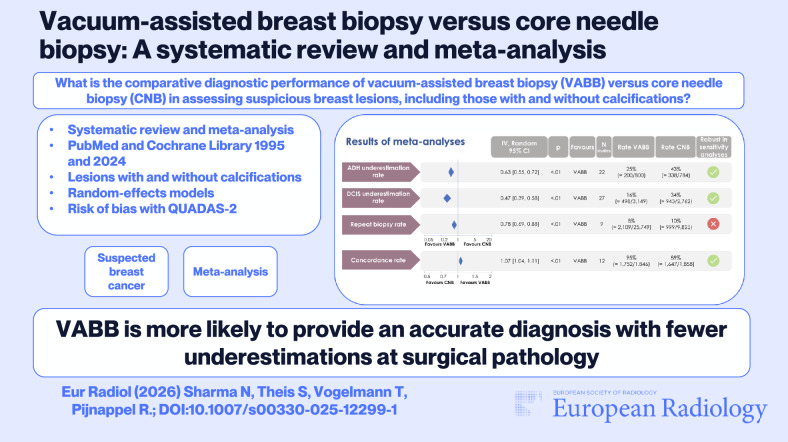

## Introduction

Approximately 2.3 million women were newly diagnosed worldwide with breast cancer in 2022 [1]. Over the last decades, the incidence of breast cancer has continuously increased by approximately 1.44% annually since 1990 [2]. Next to technological developments, this may also be related to the implementation of national breast cancer screening programs, allowing the early detection of malignant breast lesions in asymptomatic patients [3]. Patients participating in screening programs, but also symptomatic patients, first undergo diagnostic imaging using mammography, tomosynthesis, ultrasound, or MRI. When imaging results show lesions that are indeterminate, suspicious of malignancy, or malignant, patients will receive a percutaneous biopsy. Fine-needle aspiration biopsy (FNAB) was the first minimally invasive biopsy method established in the 1960s [4]. Owing to the disadvantages of FNAB, including limitations in diagnostic accuracy, core needle biopsy (CNB) became the new standard in the 1990s [4]. A hollow needle is guided in the area of concern, and small cylinders of tissue (core) are removed for histological assessment. The vacuum-assisted breast biopsy (VABB) system was developed as a modification of CNB in 1995, allowing removal of a larger amount of tissue and multiple samples from the breast by vacuum [4, 5]. Especially in very small lesions and small areas with calcifications that can be an early sign of abnormal cells developing, VABB shows a higher sensitivity in comparison to CNB [6, 7].

Although single studies exist comparing VABB and CNB, only one comparative meta-analysis was identified. Huang et al [8] included studies published until June 2016 reporting the diagnostic performance of VABB and CNB in women with breast calcifications. They found significantly lower DCIS underestimation and higher calcification retrieval with VABB compared to CNB.

Huang et al [8] provided an initial comparison between VABB and CNB, but results were based on seven studies with a maximum number of patients in the meta-analyses of 2200 and a limited set of outcomes. Our present work substantially expands this evidence base by including 60 comparative studies with up to a total number of 35,500 patients and performing meta-analyses across eight clinically relevant outcomes, allowing a detailed estimation of differential risks in women undergoing VABB vs CNB.

In contrast to previous reviews, we included the latest published data up to July 2024. Moreover, subgroup analyses were conducted exclusively for stereotactic-guided VABB and CNB, enabling a focused assessment of performance under comparable imaging conditions.

We therefore aimed to perform a systematic review and meta-analysis comparing the DCIS and ADH underestimation rate, the repeat biopsy rate, the concordance rate, the calcification retrieval rate, as well as the sensitivity, specificity, and the false-negative rate of any imaging-guided VABB to any imaging-guided CNB. ADH and DCIS underestimation were chosen as the primary outcomes of this review, as these represent the most clinically relevant and consistently reported indicators of diagnostic accuracy in percutaneous breast biopsy. Both lesions occupy critical points in the preinvasive–invasive spectrum, where diagnostic misclassification can alter treatment planning and patient management. Other potential misclassifications, such as those involving invasive carcinoma components or biomarker discordance, were insufficiently reported across studies to permit quantitative analysis.

As we defined no restrictions regarding the imaging guidance of VABB and CNB in our analyses, we further focused on the comparison of X-ray guided VABB to X-ray guided CNB.

## Materials and methods

### Literature search

A systematic review was performed searching studies in PubMed and Cochrane Library published between January 1, 1995, and July 19, 2024, with available abstracts and published in English. Search terms are available in the electronic Supplementary Material (ESM), S1. This study is reported in accordance with the Preferred Reporting Items for Systematic reviews and Meta-Analysis (PRISMA) guidelines [9, 10]. The completed PRISMA-DTA checklist has been added as ESM, S2. Our protocol was registered on INPLASY under registration number INPLASY202560074, as data extraction had already commenced at the time of registration, precluding PROSPERO eligibility.

### Eligibility criteria

Prospective and retrospective studies with a comparative design reporting original data were evaluated as eligible if, first, patients with suspected breast cancer were considered. Second, studies were included if VABB was compared to CNB. All types of imaging-guidance (ultrasound, X-ray, or MRI) and needle gauges (g) were considered. Lesions with calcifications, only consisting of calcifications, and without calcifications were included. Third, studies reporting sensitivity, specificity, false-negative rate, ADH underestimation rate, DCIS underestimation rate, repeat biopsy rate, or the concordance rate were included. Fourth, studies were required to use an appropriate reference standard, defined as surgical histopathology or patient follow-up of at least six months.

### Study selection and data extraction

Records identified through database search were first screened for eligibility based on information provided in the title and abstract, and second, using the full texts of articles. All records were independently reviewed by the same two researchers with extensive prior experience in data extraction in imaging diagnostics for suspected breast cancer. In cases of disagreement, a third reviewer—an expert with more than 15 years of experience in the field—evaluated the record, and consensus was reached through discussion. Data were extracted into a pretested spreadsheet. The spreadsheet was designed according to the checklist of the data extraction for complex meta-analysis guide [11], with the corresponding data extraction template provided in ESM, S3.

### Quality assessment

The RoB and applicability were evaluated by two reviewers independently using the Quality Assessment of Diagnostic Accuracy Studies (QUADAS)-2 tool [12]. In case of any disagreement, a third reviewer was asked for assessment, and consensus was reached by discussion. Studies were assessed for RoB regarding the dimensions (I) patient selection, (II) index test, (III) reference standard, and (IV) flow and timing. Applicability was evaluated by dimensions I to III.

### Definition of outcomes

The ADH underestimation rate refers to the percentage of patients diagnosed with ADH on VABB or CNB who had a diagnosis of DCIS or invasive cancer on their final histology report based on open surgery or on follow-up, in most studies described to be 6–12 months (= reference standard). The DCIS underestimation rate refers to the percentage of patients diagnosed with DCIS on VABB or CNB who had a diagnosis of invasive cancer on the reference standard. The repeat biopsy rate is defined as the number of repeated biopsies after the first biopsy divided by the total number of first biopsies done with either VABB or CNB. The concordance rate is defined as the number of biopsies with the same surgical histological assessment after VABB or CNB divided by the total number of surgically proven lesions. The calcification retrieval rate is defined as the number of lesions whose specimens were calcification-positive on radiography divided by the total number of biopsied lesions. Sensitivity, specificity, and false-negative rate were defined as common in diagnostic test accuracy studies [13].

### Statistical analysis

We modeled test accuracy using the bivariate random-effects (Reitsma) model. For VABB and CNB, a model was fitted to the logit-transformed sensitivity and false-positive rate, allowing for between-study heterogeneity and their covariance. To stabilize studies with zero cells, we used a symmetric Haldane–Anscombe continuity correction (+0.5 in all four cells). From the fitted models we derived (i) the summary operating point (pooled sensitivity and specificity) with delta-method 95% CIs on the probability scale, (ii) 95% confidence and 95% prediction ellipses around the summary point, (iii) area under the curve (AUC) of the hierarchical summary receiver operating characteristic (HSROC), and (iv) positive and negative likelihood ratio (LR⁺, LR⁻) and diagnostic odds ratio at the summary point (with bootstrap CIs for fixed-effect uncertainty). To compare VABB vs CNB at the HSROC summary operating points, we performed a two-degree-of-freedom Wald test on the joint difference. Pooled point sensitivities and specificities were then calculated for VABB and CNB, each along with 95% CI.

Random-effects models were used in all meta-analyses to address the expected heterogeneity between the included studies [14].

Pooled risk ratios and corresponding 95% confidence intervals (CIs) were calculated using the inverse-variance method within a random-effects model with Hartung–Knapp adjustment. Heterogeneity was assessed using the *I*² statistic with 95% CIs and τ² estimates. Prediction intervals were calculated to illustrate the expected range of effects in comparable future studies. *p* values less than 0.05 were defined as an indicator of statistical significance.

To explore potential sources of heterogeneity, mixed-effects meta-regression analyses were conducted using study-level covariates, including lesion type (calcified vs non-calcified and masses), study design (prospective vs retrospective), and region (Europe, North America, and South America) if more than ten studies were available for the outcome. The restricted maximum likelihood (REML) estimator was applied to model between-study variance. Heterogeneity explained by moderators was quantified using *R*², and residual heterogeneity was expressed as τ² and *I*². Tests of moderators were performed using the omnibus Wald test.

To test the robustness of results, sensitivity analyses were performed using the leave-one-out approach (see ESM, S4). Each study was excluded once from meta-analysis, and results were analyzed to verify whether findings depend on a single study. Studies reporting outcomes based on the same population were included once (the publication with the larger sample size).

To investigate potential publication bias, we constructed funnel plots to graphically represent the relationship between study effect sizes and their precision. Asymmetry in these plots may suggest the presence of bias. Additionally, Egger’s regression analysis was performed to statistically evaluate the correlation between effect sizes and their standard errors, providing a quantitative measure of publication bias.

The analyses were conducted using Review Manager (for all outcomes reporting relative risk (RR)) and Microsoft Excel (for sensitivity and specificity), as well as R 4.5.2 for regression analyses.

Besides comparing any imaging-guided VABB to any imaging-guided CNB (including ultrasound, X-ray, or MRI-guided VABB and/or CNB), we further analyzed the outcomes, only including studies reporting results for X-ray-guided VABB compared to X-ray-guided CNB. Additionally, we analyzed the outcomes of studies comparing X-ray guided VABB to any imaging-guided CNB (given in the ESM, S5).

## Results

### Study selection

**Fig. 1:**
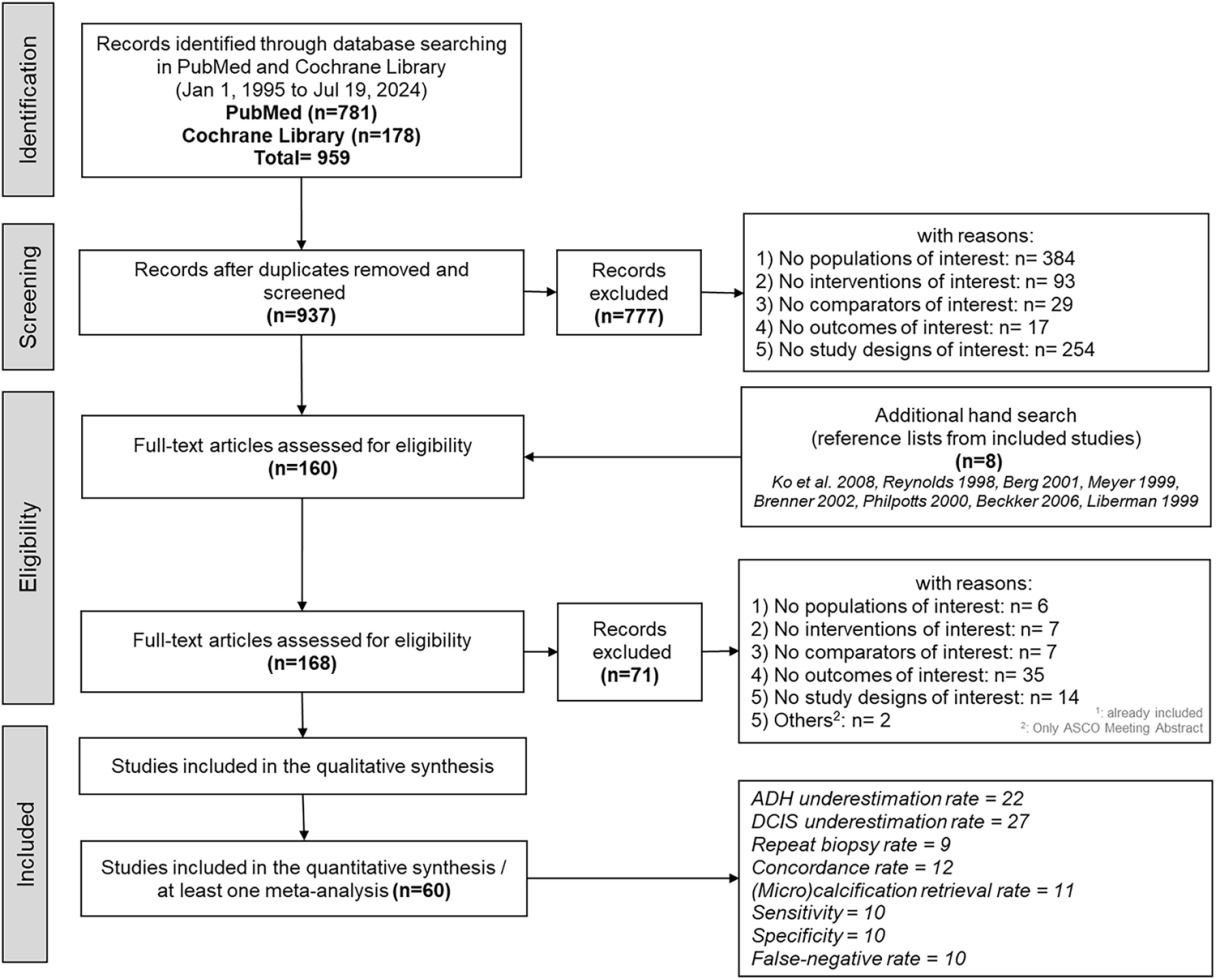
PRISMA flowchart of studies describing the process of selecting studies included in meta-analysis. ADH atypical ductal hyperplasia; DCIS ductal carcinoma in situ

Nine hundred fifty-nine records were identified in PubMed (*n* = 781) and Cochrane Library (*n* = 178). Twenty-two duplicates were removed, leaving 937 records for the title and abstract screening. Therefore, 777 records were excluded. 8 studies were added by hand search to the full text screening. One hundred sixty-eight records were assessed for eligibility in full text. Sixty records were included in at least one meta-analysis. The selection process is shown in Fig. 1.

### Study characteristics

Table 1 summarizes characteristics of studies comparing VABB to CNB. Of the 60 studies included, 56 (93%) used a retrospective design, while two were randomized and two were prospective. Approximately half of the studies were conducted in North America, one-fifth in Europe, and one-third in Asia. VABB was mostly performed under X-ray guidance using 11-g needles, followed by ultrasound guidance using 14-g needles. CNB was mostly performed under ultrasound guidance using 14-g needles followed by X-ray guidance.

**Table 1:** Summary of included studies

						VABB			CNB	
Author	Year	Study design	Nation	Lesion types	Reference standard	Imaging-guidance	Needle gauge	Device	Imaging-guidance	Needle gauge
Badan [22]	2016	R	Brazil	Calc	Surg.	X-ray	9	ATEC	X-ray	12
Bae [23]	2015	R	South Korea	Calc	Surg./FU	US/X-ray	8, 11	Mammatome	US	14
Becker [24]	2006	R	Canada	Masses and calc	Surg./FU	NA	11	Mammatome	NA	14
Berg [25]	2001	R	U.S.	Calc	Surg.	X-ray	11	Mammatome	X-ray	14
Bertani [26]	2020	R	Italy	Calc	Surg.	X-ray	9	Eviva	US	14
Brenner [27]	2000	R	U.S.	Masses and calc	Surg./FU	US/X-ray	11, 14	Mammatome	US/X-ray	12, 14
Bundred [18]	2016	RCT	UK	Calc	Surg.	X-ray	11	Mammatome	X-ray	14
Burbank [5]	1997	R	U.S.	Masses and calc	Surg.	X-ray	14	Mammatome	X-ray	14
Cho [28]	2005	R	South Korea	Masses and calc	Surg./FU	US	11	Mammatome	US	14
Ciatto [6]	2007	R	Italy	Calc	Surg./FU	US/X-ray	11	Mammatome	US/X-ray	14
Darling [29]	2000	R	U.S.	Masses and calc	Surg.	X-ray	11, 14	Mammatome	US/X-ray	14
Doria [30]	2018	R	Brazil	Masses and calc	Surg.	US/X-ray /MRI	11, 14	Mammatome	US	14, 16
Elsharkawy [31]	2020	R	Germany	Masses and calc	Surg.	X-ray	8, 10	Mammatome, Vacora	US	14
Fuentes [32]	2019	R	Venezuela	Masses and calc	Surg.	US/X-ray	7, 8, 9, 11, 12, 13	Mammatome, ATEC	US/X-ray	NA
Grady [15]	2017	R	U.S	NA	NA	US	7–14	Mammatome, ATEC, Celero, EnCore Vacora, Finesse	US	14
Hsieh [33]	2023	R	Taiwan	Masses and calc	Surg.	X-ray	10	Encor	US	16
Huang [34]	2011	R	Taiwan	Calc	Surg.	X-ray	9, 10	ATEC, Vacora	X-ray	14
Jackman [35]	2001	R	U.S	Masses and calc	Surg.	X-ray	11, 14	Mammatome	X-ray	14
Jackman [36]	1997	R	U.S	Masses and calc	Surg.	X-ray	14	NA	X-ray	14
Jackman [37]	2006	R	U.S.	Calc	Surg./FU	X-ray	11, 14	Mammatome	X-ray	14
Kil [38]	2008	R	South Korea	Masses and calc	Surg.	US/X-ray	11	Mammatome	US	14
Kim [39]	2012	R	South Korea	Masses and calc	Surg.	US	8, 11	Mammatome	US	14
Ko [40]	2008	R	South Korea	Masses and calc	Surg.	US	11	Mammatome	US	14
La Forgia [16]	2020	R	Italy	Masses and calc	Surg.	US	13	Mammatome, Leica	US	14, 15, 16
Lacambra [41]	2012	R	China, Singapore	Masses and calc	Surg./FU	NA	NA	Mammatome, EnCor	NA	NA
Lee [42]	2013	R	South Korea	Masses and calc	Surg.	NA	NA	NA	NA	NA
Liberman [43]	2001	R	U.S.	Calc	Surg.	X-ray	11, 14	Mammatome	X-ray	14
Liberman [44]	2000	R	U.S.	Masses and calc	Surg.	X-ray	11, 14	Mammatome	US/X-ray	14
Liberman [45]	2001	R	U.S.	Masses and calc	Surg./FU	X-ray	11, 14	Mammatome	X-ray	14
Londero [46]	2011	R	Italy	Masses and calc	Surg./FU	X-ray	11	Mammatome	US	14
Mannu [47]	2019	R	Netherlands	Masses and calc	Surg.	US/X-ray	9	NA	US/X-ray	14
Marques [48]	2019	R	Brazil	Masses and calc	Surg.	US/X-ray	NA	NA	US/X-ray	NA
Meyer [49]	1997	R	U.S.	Calc	Surg./FU	US/X-ray	14	Mammatome	US/X-ray	14
Middleton [50]	2003	R	U.S.	Masses and calc	Surg	X-ray	11	Mammatome	US	14, 18–20
Oktay [51]	2023	R	Turkey	Masses and calc	Surg./FU	X-ray	9–12	NA	US	14–16
Park [52]	2022	R	South Korea	Masses and calc	Surg./FU	US/X-ray	8–11	Mammatome, ATEC	US	14
Philpotts [53]	2003	R	U.S.	NA	Surg.	US	11	Mammatome	US	14
Philpotts [54]	1999	R	U.S.	Masses and calc	Surg./FU	NA	11	Mammatome	X-ray	14
Poole [55]	2015	R	U.S.	Masses and calc	Surg.	US/X-ray /MRI	8–12	Mammatome, ATEC, Celero, Eviva, EnCore, Vacora	US/X-ray/MRI	Majority: 14
Povoski [56]	2011	R	U.S.	NA	Surg./FU	US	8	Mammatome	US	14
Rageth [57]	2019	R	Switzerland	Masses and calc	Surg.	US/X-ray/MRI	7, 9, 11	Mammatome, Eviva, EnCore	US	14
Reynolds [58]	1998	R	U.S.	Calc	NA	X-ray	11	Mammatome	X-ray	14
Seely [59]	2017	R	Canada	Masses and calc	Surg./FU	US/X-ray	10–12	Mammatome, EnCor, SenoCor	US/X-ray	14
Seo [60]	2017	P	South Korea	Calc	Surg.	US	13	Mammatome	US	14
Sheng [61]	2020	R	China	NA	Surg.	US	10	Mammatome	US	14
Shin [62]	2008	R	South Korea	Masses and calc	Surg./FU	US	8, 11	Mammatome	US	NA
Sim [63]	2015	R	UK	Masses and calc	Surg.	US/X-ray	NA	Mammatome, Vacora, EnCore	NA	14
Soo [64]	1999	R	U.S.	Masses	Surg.	X-ray	14	Mammatome	X-ray	14
Suh [65]	2012	R	South Korea	Masses and calc	Surg.	US	8, 11	Mammatome	US	14
Szynglarewicz [66]	2015	P	Poland	Masses and calc	Surg.	US	10, 11	Mammatome, EnCore,	US	14
Tian [67]	2024	R	China	Masses	Surg.	US	NA	Mammatome	US	NA
Tothova [68]	2013	R	Slovakia	Masses and calc	Surg.	X-ray	9, 11	Mammatome, ATEC	X-ray	14
Velanovich [69]	1999	R	U.S.	Masses and calc	Surg./FU	X-ray	11	Mammatome	X-ray	14
Willers [70]	2023	R	Belgium	NA	Surg.	NA	NA	NA	NA	NA
Won [71]	1999	R	U.S.	Masses and calc	Surg.	X-ray	11	Mammatome	X-ray	14
Yashima [72]	2023	R	Japan	Masses and calc	Surg./FU	US	12	Celero	US	16
Zannis [73]	1998	R	U.S.	Masses and calc	Surg./FU	X-ray	11, 14	Mammatome	X-ray	14
Zhang [7]	2023	RCT	China	Masses and calc	Surg.	US	10	Mammatome	US	14
Zhao [74]	2002	R	U.S.	Masses and calc	Surg.	X-ray	11, 14	Mammatome	NA	14
Zou [75]	2019	R	China	Masses	Surg.	US	8, 10	Mammatome	US	14

*R* retrospective, *P* prospective, *RCT* randomized controlled trial, *U.S.* United States, *UK* United Kingdom, *calc* calcifications, *Surg.* surgery, *FU* follow-up, *US* ultrasound, *MRI* magnetic resonance imaging, *NA* not available *CNB* core needle biopsy, *VABB* vacuum-assisted breast biopsy

### Quality assessment

**Fig. 2:**
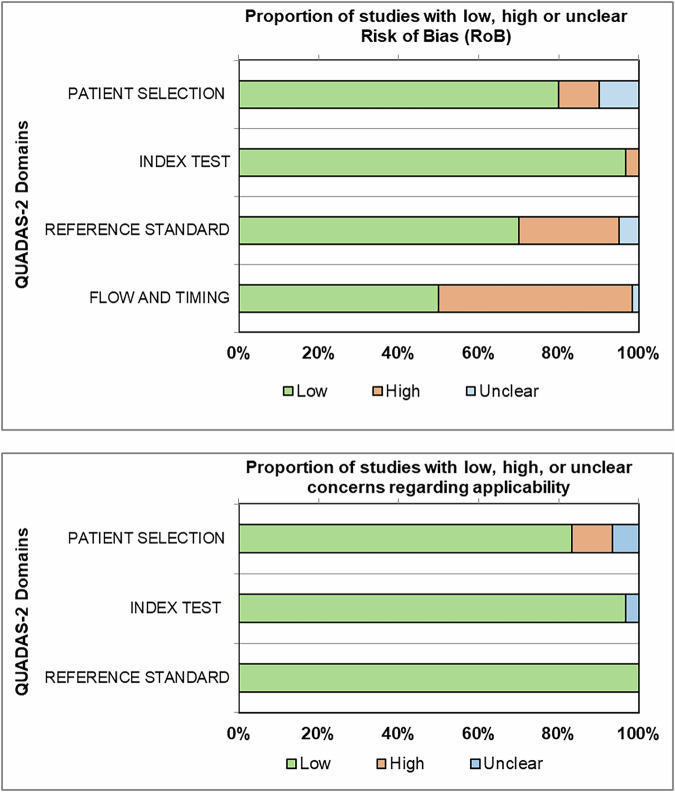
Quality assessment of included studies using QUADAS-2

Figure 2 shows the RoB and applicability assessment for the 60 studies included in at least one meta-analysis. Approximately one-half of the studies were evaluated as having a high RoB in the domain ‘flow and timing’, as not every woman received the same reference test after VABB or CNB. Low RoB in that domain would require that all patients, including patients with benign lesions found at VABB or CNB, subsequently undergo surgical excision. Studies in patients with benign lesions who only had imaging follow-up were judged to have a high RoB in the domain *‘*flow and timing’. A detailed study-level QUADAS-2 assessment is provided as ESM, S6, reporting individual domain-level risk-of-bias and applicability judgments for each included study.

### Synthesis of results

#### ADH and DCIS underestimation rates

**Fig. 3:**
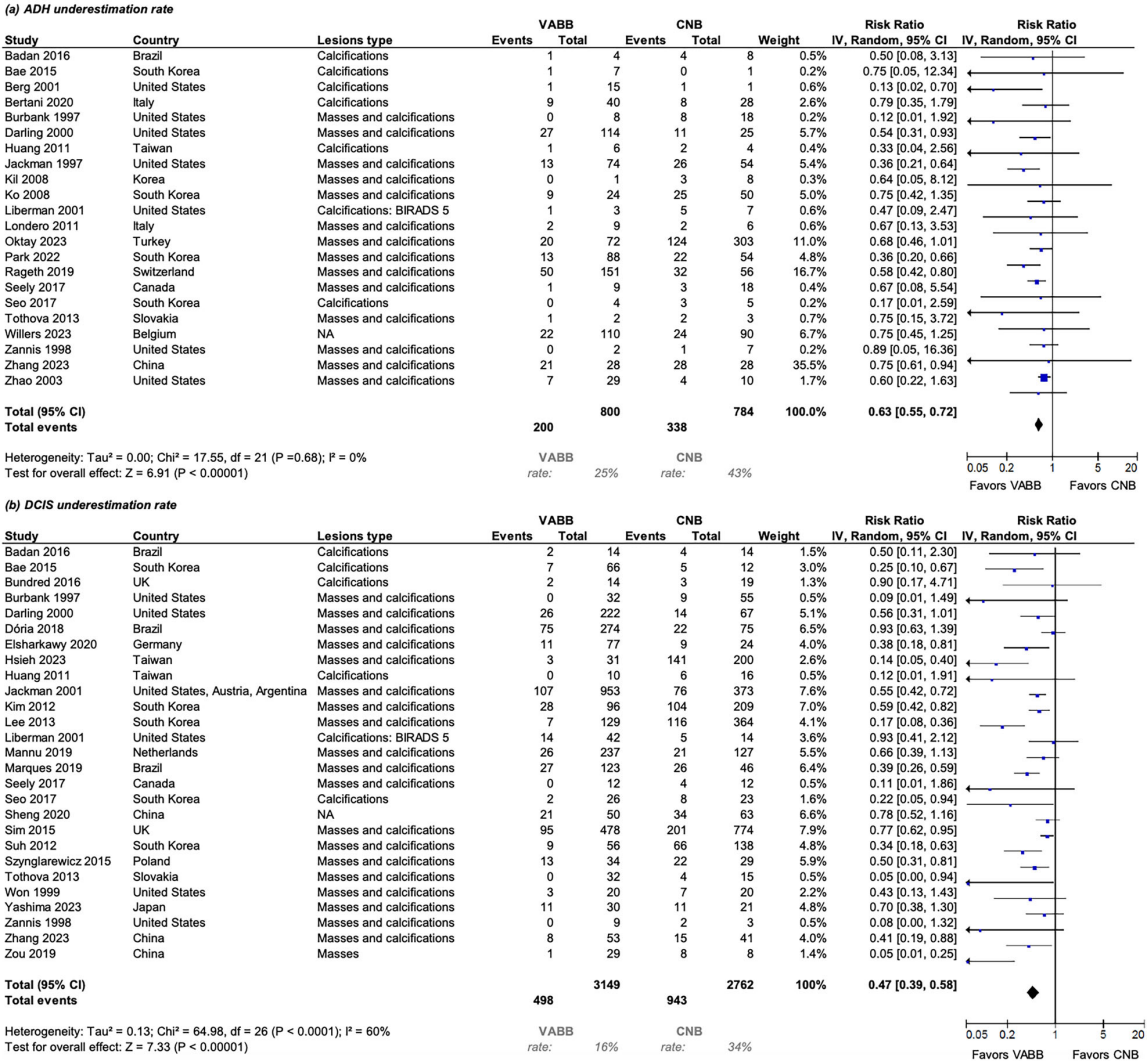
Forest plots for **a** ADH and **b** DCIS underestimation rate in patients biopsied with VABB compared to CNB. Squares with horizontal lines represent individual study estimates and 95% CI. Diamond represents the pooled estimate and 95% CI. *ADH* atypical ductal hyperplasia, *CNB* core needle biopsy, *DCIS* ductal carcinoma in situ, *VABB* vacuum-assisted breast biopsy

Twenty-two studies, including a total of 1584 lesions examined with VABB or CNB, were included in the meta-analysis of the ADH underestimation rate. The ADH underestimation rate was estimated to be significantly lower when using VABB compared to CNB (Pooled risk ratio derived from random-effects model [RRREM]: 0.63, 95% CI: 0.55–0.72, *p* < 0.01), see Fig. 3a. Between-study heterogeneity was negligible (*I*² = 0%), and the 95% prediction interval (0.55–0.72) suggested consistent findings across settings.

In a meta-regression model including lesion type, study design, and geographic region as covariates, 53.5% of the between-study heterogeneity in ADH underestimation was explained (*R*² = 53.45%). Residual heterogeneity was low (τ² = 0.01, *I*² = 5.0%). None of the examined covariates reached statistical significance (omnibus F[5, 16] = 1.91, *p* = 0.15), indicating that differences in lesion type, study design, or geographic distribution did not materially influence the observed effect. The model fit was adequate (logLik = −13.41, AIC = 40.82, BIC = 46.23). Funnel plots for all outcomes appeared symmetric, and Egger’s test showed no evidence of small-study bias (intercept = 0.24, *p* = 0.63) (ESM, S7). Sensitivity analyses demonstrated robustness regarding statistical significance (ESM, S4).

Twenty-seven studies, including a total of 5911 lesions examined with VABB or CNB, were included in the meta-analysis of the DCIS underestimation rate. In 27 studies evaluating DCIS underestimation, VABB was associated with a significantly lower risk compared with CNB (RR = 0.48, 95% CI: 0.39–0.59; *I*² = 59%), see Fig. 3b. The 95% prediction interval (0.23–0.98) indicates that the VABB advantage may vary across settings.

In the meta-regression model included, residual heterogeneity was substantial (τ² = 0.2266, SE = 0.1246; *I*² = 65.9%), indicating considerable variability among studies. The heterogeneity accounted for by covariates (lesion type, region, study design) was negligible (*R*² = 0.0%). The test for residual heterogeneity remained significant (Q_E[21] = 55.77, *p* < 0.0001), suggesting that unmeasured factors may contribute to between-study differences. The model’s overall fit was adequate (logLik = −25.21, AIC = 64.43, BIC = 71.74). The test of moderators was not significant (F[5, 21] = 0.58, *p* = 0.71), indicating that the included covariates did not explain heterogeneity in DCIS underestimation. The intercept term was significant (β = −1.0380, SE = 0.4842, *p* = 0.0439, 95% CI: −2.0450 to −0.0311), confirming an overall lower underestimation rate with VABB compared to CNB. None of the examined moderators—lesion type, geographic region, or study design—showed significant associations (all *p* > 0.15).

Funnel-plot asymmetry was evident (Egger’s *p* = 0.0003; Begg’s *p* = 0.0001), suggesting possible small-study effects (ESM, S7). Sensitivity analyses demonstrated robustness regarding statistical significance (ESM, S4).

#### Repeat biopsy rate, concordance rate, and calcification retrieval rate

**Fig. 4:**
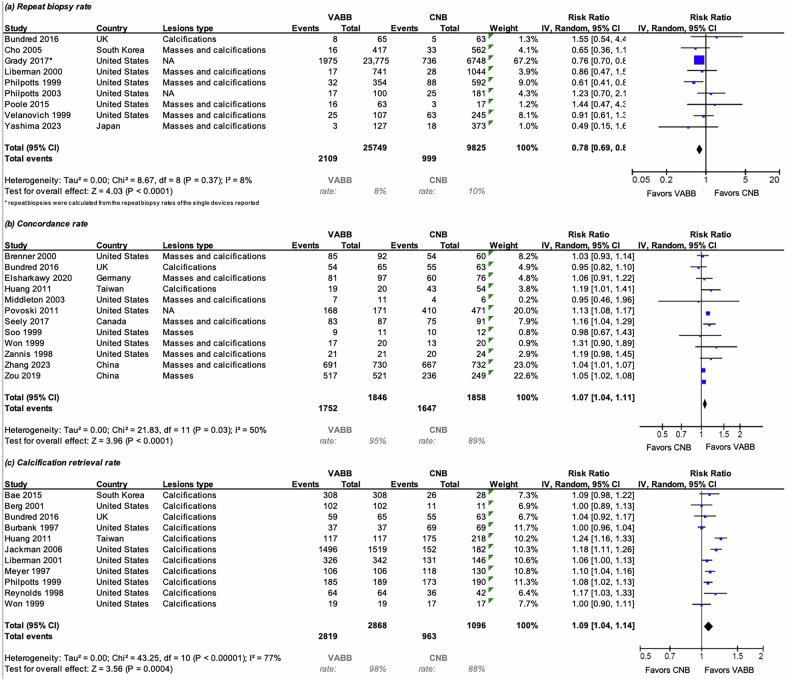
Forest plots for **a** repeat biopsy rate, **b** concordance rate, and **c** calcification retrieval rate in patients biopsied with VABB compared to CNB. Squares with horizontal lines represent individual study estimates and 95% CI. Diamond represents the pooled estimate and 95% CI. CNB, core needle biopsy, VABB, vacuum-assisted breast biopsy

9 studies, including a total of 35,574 lesions examined with VABB or CNB, were included in the meta-analysis of repeat biopsy rate. Across nine studies, repeat biopsy rates were significantly lower following VABB than CNB (RR = 0.78, 95% CI: 0.69–0.88; *I*² = 8%). See Fig. 4, a. The 95% prediction interval (0.66–0.93) suggests consistent benefit across settings. No funnel-plot asymmetry was detected (Egger’s *p* = 0.34; Begg’s *p* = 0.61). Statistical significance was lost when excluding study data from Grady et al [15], but the effect direction was still in favor of VABB (RRREM: 0.83, 95% CI: 0.66–1.05, *p* = 0.13), see ESM, S4. In 12 studies, VABB demonstrated a higher histopathologic concordance with surgical specimens than CNB (RR = 1.07, 95% CI: 1.04–1.11; *I*² = 51%), see Fig. 4b. The 95% prediction interval (0.99–1.16) suggests a consistent advantage across most settings. In the meta-regression model included, no residual heterogeneity was detected (τ² = 0.0000, SE = 0.0015; *I*² = 0%), indicating that almost all observed variability was explained by sampling error or model covariates. The amount of heterogeneity accounted for by moderators was complete (*R*² = 100%). The test for residual heterogeneity was not significant (Q_E[6] = 5.13, *p* = 0.53), confirming model adequacy. The overall model fit was satisfactory (logLik = 5.34, AIC = 3.32, BIC = 1.87). The test of moderators approached statistical significance (F[5, 6] = 4.00, *p* = 0.061), suggesting a possible influence of study characteristics. The intercept term was marginally non-significant (β = 0.1687, SE = 0.0817, *p* = 0.0846, 95% CI: −0.0313 to 0.3686), indicating a weak positive association favoring VABB in overall concordance. Among the covariates, only studies conducted in North America showed a significant positive effect (β = 0.0691, *p* = 0.0183, 95% CI: 0.0165–0.1218), while lesion type, European region, and retrospective design were not significantly associated with concordance (all *p* > 0.12). Funnel-plot asymmetry was absent (Egger’s *p* = 0.43; Begg’s *p* = 0.64), ESM, S7. Sensitivity analyses demonstrated robustness regarding statistical significance (ESM, S4).

Across 11 studies, calcification retrieval was consistently higher with VABB than with CNB. On the logit scale, the pooled difference was 1.78 (95% CI: 0.95–2.61), corresponding to an approximate 71% higher retrieval probability for VABB, see Fig. 4c. Heterogeneity was moderate (*I*² = 64%). Funnel plots appeared symmetric, and both Egger’s (*p* = 0.94) and Begg’s (p = 0.65) tests indicated no publication bias, ESM, S7. In a meta-regression model, residual heterogeneity was moderate to substantial (τ² = 0.0032, SE = 0.0023; *I*² = 73.7%). The amount of heterogeneity accounted for by study-level covariates (region: Europe, North America vs Asia) was 17.9% (*R*² = 17.87%). The test for residual heterogeneity remained significant (Q_E[8] = 29.72, *p* = 0.0002), while the overall model fit was adequate (logLik = 9.82, AIC = −11.65, BIC = −11.33). The test of moderators was not statistically significant (F[2, 8] = 1.36, *p* = 0.31), indicating that regional differences did not explain the observed heterogeneity. The intercept term was significant (β = 0.1566, SE = 0.0504, *p* = 0.0145, 95% CI: 0.0405–0.2728), suggesting an overall positive association favoring VABB in terms of microcalcification retrieval rate. The regional moderators (Europe and North America) were not significant (β = −0.1177, *p* = 0.27; β = −0.0874, *p* = 0.16, respectively). Sensitivity analyses demonstrated robustness regarding statistical significance (ESM, S4).

#### Sensitivity, specificity, and false-negative rate

**Fig. 5:**
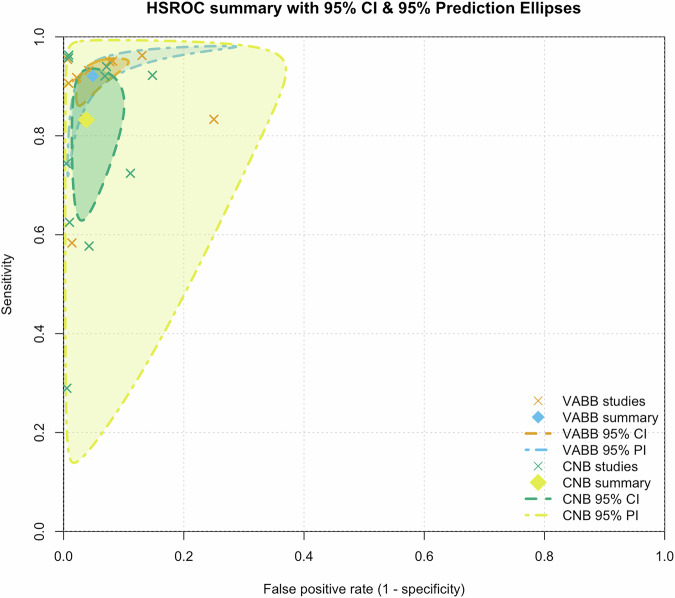
HSROC curves comparing VABB and CNB for the diagnosis of suspicious breast lesions. The plot displays the summary operating points (diamonds) with 95% confidence (CI) and 95% prediction intervals (PI) for both VABB (blue) and CNB (yellow). Individual study estimates of sensitivity and false-positive rate are shown as crosses. The HSROC model was fitted using the Reitsma bivariate random-effects approach with restricted maximum likelihood (REML) estimation. Both techniques demonstrated high overall diagnostic performance with overlapping confidence regions, indicating comparable accuracy across studies. CNB, core needle biopsy; HSROC, hierarchical summary receiver operating characteristic; VABB, vacuum-assisted breast biopsy

**Fig. 6:**
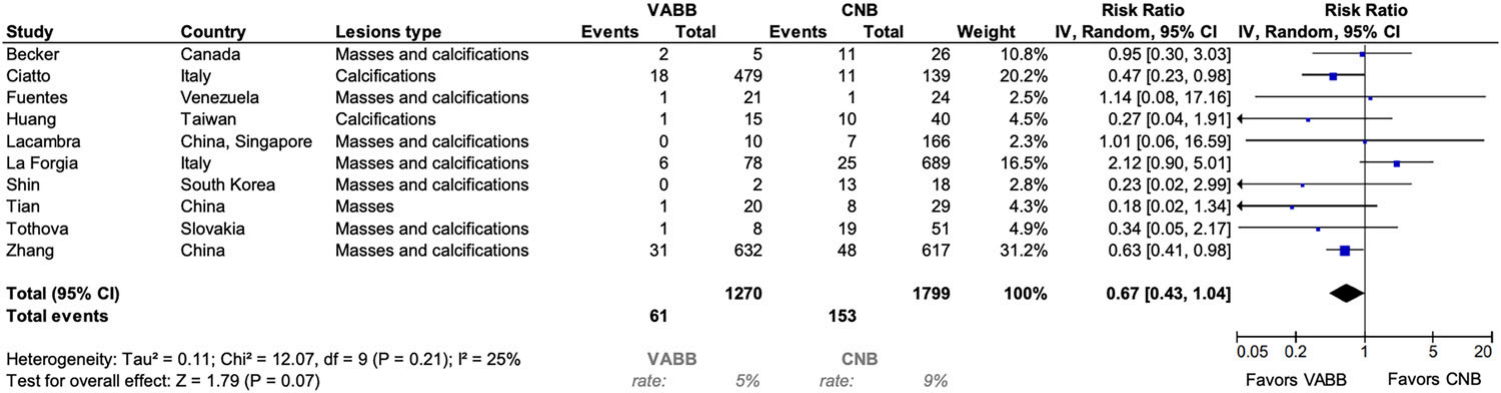
Forest plots for the false-negative rate in patients biopsied with VABB compared to CNB. Squares with horizontal lines represent individual study estimates and 95% CI. Diamond represents the pooled estimate and 95% CI. CNB, core needle biopsy; VABB, vacuum-assisted breast biopsy

The sensitivities and specificities calculated for VABB and CNB are given in Fig. 5. 10 studies reported full sets of true-positives, false-positives, false-negatives, and true-negatives and were included in the meta-analyses for sensitivity and specificity. These studies included a total of 4,822 lesions examined with VABB or CNB. The sensitivity for VABB was calculated to be 94% (95% CI: 92–97%) compared to a sensitivity of 78% for CNB (95% CI: 70–86%). The specificity for VABB was calculated to be 97% (95% CI: 92–100%), equal to that of CNB (95% CI: 95–100%) (Fig. 6).

Using separate HSROC (Reitsma) fits for VABB and CNB, the summary operating point for VABB was sensitivity 0.921 (95% CI: 0.875–0.951) and specificity 0.951 (95% CI: 0.907–0.975); the corresponding AUC was 0.971. For CNB, the summary operating point was sensitivity 0.832 (95% CI: 0.677–0.922) and specificity 0.962 (95% CI: 0.917–0.983) with AUC 0.970. At the summary points this implies, for VABB, LR⁺ 18.9 (95% CI: 10.0–35.4), LR⁻ 0.08 (95% CI: 0.05–0.13), and DOR 227.8 (95% CI: 143.3–358.7); for CNB, LR⁺ 21.9 (95% CI: 10.2–47.8), LR⁻ 0.17 (95% CI: 0.08–0.34), and DOR 125.7 (95% CI: 44.1–357.5). Between-study heterogeneity was moderate for VABB (τ²_sens ≈ 0.32; τ²_spec ≈ 0.59) and substantial for CNB (τ²_sens ≈ 1.77; τ²_spec ≈ 1.03). A joint Wald test comparing VABB vs CNB at the summary operating points was not statistically significant (χ² = 2.83, df = 2, *p* = 0.243), consistent with the overlapping confidence ellipses.

10 studies, including a total of 3069 lesions examined with VABB or CNB, were included in the meta-analysis of the false-negative rate. The false-negative rate was estimated to be lower (but not statistically significant) when using VABB compared to CNB (RRREM: 0.67, 95% CI: 0.43–1.04, *p* = 0.07). Sensitivity analyses showed a statistically significantly lower risk of cancers misdiagnosed as benign with VABB as with CNB when the study results of La Forgia et al [16] were excluded (RRREM: 0.57, 95% CI: 0.41–0.79, *p* < 0.01), see ESM, S4.

#### X-ray guided VABB vs X-ray guided CNB

The pooled outcomes of studies comparing X-ray guided VABB to X-ray guided CNB, as well as the results of comparing any imaging-guided biopsies, are given in Table 2. The risk of ADH underestimation was estimated to be significantly lower when comparing any imaging-guided VABB to any imaging-guided CNB (RRREM: 0.63, 95% CI: 0.55–0.72, *p* < 0.01). When only including studies that compared X-ray guided VABB to X-ray guided CNB, the risk ratio of ADH underestimation (9 studies) decreased further (RRREM: 0.33, 95% CI: 0.24–0.46, *p* < 0.01). The risk of a lower DCIS underestimation and a higher calcification retrieval with any imaging-guided VABB compared to any imaging-guided CNB did not change when only including X-ray guided VABB compared to X-ray guided CNB, but statistically significant risk advantages in favor of any imaging-guided VABB compared to any imaging-guided CNB for repeated biopsies and concordance were lost when only including studies comparing X-ray guided VABB to X-ray guided CNB.

**Table 2:** Results of the subgroup analyses for all outcomes considered

(a) Results of including all studies (comparing any imaging-guided VABB to any imaging-guided CNB) and comparing X-ray guided VABB to X-ray guided CNB: ADH underestimation rate, DCIS underestimation rate, repeat biopsy rate, concordance rate, calcification retrieval rate, and false negative rate			
	No. studies	Risk ratio (95% CI) using REM, *p* value	Favor
ADH underestimation rate	22	0.63 (0.55–0.72), *p* < 0.01	VABB
X-ray guided VABB vs X-ray guided CNB	9	0.33 (0.24–0.46), *p* < 0.01	VABB
DCIS underestimation rate	27	0.47 (0.39–0.58), *p* < 0.01	VABB
X-ray guided VABB vs X-ray guided CNB	10	0.55 (0.42–0.72), *p* < 0.01	VABB
Repeat biopsy rate	9	0.78 (0.69–0.88), *p* < 0.01	VABB
X-ray guided VABB vs X-ray guided CNB	2	0.97 (0.67–1.42), *p* = 0.88	-
Concordance rate	12	1.07 (1.04–1.11), *p* < 0.01	VABB
X-ray guided VABB vs X-ray guided CNB	5	1.10 (0.97–1.25), *p* = 0.13	-
Calcification retrieval rate	11	1.09 (1.04–1.14), *p* < 0.01	VABB
X-ray guided VABB vs X-ray guided CNB	8	1.01 (1.01–1.16), *p* = 0.02	VABB
False negative rate	10	0.67 (0.43–1.04), *p* = 0.07	-
X-ray guided VABB vs X-ray guided CNB	2	0.30 (0.08–1.17), *p* = 0.08	-
**(b) Results of including all studies (comparing any imaging-guided VABB to any imaging-guided CNB) and comparing X-ray guided VABB to X-ray guided CNB: sensitivity and specificity**			
	**No. studies**	**Pooled values (95% CI) using REM**	
Sensitivity	10	VABB: 0.94 (0.92–0.97) CNB: 0.78 (0.70–0.86)	
X-ray guided VABB vs X-ray guided CNB	2	xVABB: 0.91 (0.80–1.00) xCNB: 0.68 (0.56–0.80)	
Specificity	10	VABB: 0.97 (0.92–1.00) CNB: 097 (0.95–1.00)	
X-ray guided VABB vs X-ray guided CNB	2	xVABB: 1.00 (0.97–1.00) xCNB: 1.00 (0.99–1.00)	

*ADH* atypical ductal hyperplasia, *DCIS* ductal carcinoma in situ, *REM* random effects model, *VABB* vacuum-assisted breast biopsy, *CNB* core needle biopsy, *xVABB* X-ray guided VABB, *xCNB* X-ray guided CNB

## Discussion

In this meta-analysis of 60 studies, VABB consistently outperformed core CNB across several diagnostic outcomes. VABB demonstrated significantly lower underestimation rates for ADH and DCIS, fewer repeat biopsies, and higher histologic concordance and calcification retrieval rates. These findings indicate that VABB provides a more accurate and efficient diagnostic pathway for suspicious breast lesions.

We found that patients biopsied with any imaging-guided VABB had a 53% decreased risk of DCIS underestimation compared to patients biopsied with any imaging-guided CNB. Huang et al [8] also reported a significantly lower rate of DCIS underestimation with VABB, including five studies. DCIS is typically treated surgically by lumpectomy or mastectomy. A sentinel lymph node biopsy (SLNB) is not needed with lumpectomy, but is performed with mastectomy [17]. The surgical management of invasive cancers is the same as with DCIS, but SLNB is always done. When DCIS is underestimated and diagnosed as invasive cancer at lumpectomy, a second operation is needed for SLNB, which may incur a delay in treatment while awaiting histology to determine if nodes are negative or positive. Patients biopsied with any imaging-guided VABB compared to any imaging-guided CNB had a 37% decreased risk of ADH underestimation. Results were also robust in sensitivity analyses. A further decrease to a 67% lower risk of ADH underestimation was seen when using X-ray guided VABB compared to X-ray guided CNB. ADH underestimation may also be associated with treatment delay due to the need for a second operation to assess margins. Additionally, and probably more relevant, is the psychological impact that misdiagnoses have on women. Discomfort, anxiety, but also a worse quality of life and increased breast pain are often related to breast biopsy [18]. It has previously been shown [19] that women were more likely to return to breast cancer screening after a true-negative result than after a false-positive recall for additional imaging. Therefore, fewer misdiagnoses by VABB may reduce wrong expectations based on incorrect diagnoses and have an influence on women’s confidence in healthcare and treatment adherence.

The diagnostic advantages shown with VABB may result from the significantly higher calcification retrieval rate given with VABB (RR: 1.09). This is also reflected by a higher sensitivity with any imaging-guided VABB (95%) compared to any imaging-guided CNB (78%). Further, a 7% increased chance that the surgical histology will match the biopsy result with any imaging-guided VABB compared to any imaging-guided CNB strengthened the diagnostic advantages. Calcification retrieval rates and concordance rates are related to the number of biopsy specimens taken [20]. VABB and CNB highly depend on the lesion characteristics. Thus, results in favor of VABB may result from a larger specimen volume, as with CNB. As only half of the studies included reported the number of specimens taken, we were not able to stratify the results.

Additionally, we found a 22% decreased risk of receiving a repeat biopsy in patients receiving any imaging-guided VABB. Assuming that a second biopsy for further diagnosis could be avoided in every fifth woman with VABB would have a significant impact on the healthcare resource utilization, as every specimen needs to go to pathology and be discussed at the multidisciplinary meeting. Further, avoiding second diagnostic biopsies would mean that women can start treatment in a timely manner. The National Health Service (NHS) England, for example, defines a 62-day treatment standard in their modernized cancer waiting time standards from 1st October 2023 [21]. Using VABB instead of CNB may help increase the percentage of patients reaching the 62-day target.

The following limitations need to be considered: First, the included studies are heterogeneous. Some studies focused on calcifications, whereas others also included masses, and two different reference standards were used (histological results from surgical excision or non-standardized follow-up). Studies from different countries under different conditions were included, like differences in the training of the radiologists or procedure standards. Moderate to high heterogeneity was observed across most analyses, largely reflecting variations in imaging guidance, reference standards, and biopsy protocols. Despite this, sensitivity analyses yielded consistent directionality of effects, supporting the robustness of our conclusions. Risk of bias (RoB) was low in two-thirds of studies, although the domain of flow and timing remained a frequent source of concern due to incomplete verification.

Second, of the 60 studies included, only 2 had a randomized design. Thus, the observed results may result from differences between the radiologists performing the biopsy who choose different biopsy devices, guiding methods, or needle gauges with respect to the radiological characteristics, but also on patient characteristics. More randomized and prospective studies could help reduce these uncertainties. Third, the number of samples taken with VABB are assumed to be higher than the number of cores by CNB. A larger proportion of studies reporting details of the biopsy methods would facilitate a comparison of studies. While our results consistently favored VABB over CNB across most outcomes, several factors warrant cautious interpretation: VABB was most frequently performed under X-ray or tomosynthesis guidance, whereas CNB was predominantly ultrasound-guided. The superior lesion targeting and sampling accuracy of X-ray-guided procedures may therefore account for part of the observed advantage attributed to VABB. In a subgroup analysis limited to studies using comparable imaging guidance (e.g., X-ray-guided VABB vs X-ray-guided CNB), several differences between techniques were no longer statistically significant. This finding indicates that the superiority of VABB may be context-dependent rather than absolute, reflecting both technical and methodological heterogeneity across studies.

This systematic review and meta-analysis highlight the key differences between VABB vs CNB as a first-line diagnostic tool. VABB is more likely to provide a definitive diagnosis with fewer underestimations at pathology that can support timely treatment of breast cancer, as well as high-risk lesions, and reduce upgrade rates to cancer at the time of surgery (diagnostic or therapeutic). In this current climate, there are significant challenges facing the breast imaging community in terms of workforce shortages and pressures to deliver nationally set targets. However, translating these findings into clinical practice requires consideration of practical and economic factors. VABB should be prioritized as a first-line diagnostic procedure for non-palpable lesions, particularly microcalcifications or architectural distortions detected on mammography or tomosynthesis, where representative sampling to obtain the optimal diagnostic accuracy is most critical. CNB remains an efficient option for palpable or ultrasound-visible lesions, especially in settings where VABB devices or expertise are limited. The broader implementation of VABB will depend on institutional resources and cost-effectiveness analyses.

## Supplementary information

ELECTRONIC SUPPLEMENTARY MATERIAL

## Abbreviations

ADH: Atypical ductal hyperplasia
AUC: Area under the curve
CI: Confidence interval
CNB: Core needle biopsy
DCIS: Ductal carcinoma in situ
ESM: Electronic supplementary material
FNAB: Fine-needle aspiration biopsy
HSROC: Hierarchical summary receiver operating characteristic
LR: Likelihood ratio
PRISMA: Preferred reporting items for systematic reviews and meta-analysis
QUADAS: Quality assessment of diagnostic accuracy studies
REM: Random effect models
RoB: Risk of Bias
RR: Relative risk
RRREM: Pooled risk ratio derived from random-effects model
SLNB: Sentinel lymph node biopsy
VABB: Vacuum-assisted breast biopsy
