# Supplementary material for: Vacuum-assisted breast biopsy vs core needle biopsy: a systematic review and meta-analysis
Source: Eur Radiol. 2026 Jan 19;36(6):4449–66. doi: 10.1007/s00330-025-12299-1 (PMC13212802; doi:10.1007/s00330-025-12299-1)
Supplement: Supplementary file 1 — ELECTRONIC SUPPLEMENTARY MATERIAL [file 330_2025_12299_MOESM1_ESM.pdf]

Vacuum-assisted breast biopsy versus core needle biopsy: A Systematic Review and Meta-Analysis

ELECTRONIC SUPPLEMENTARY MATERIAL

S1: Search strategies PubMed and Cochrane Library

Search strategy - PubMed

(((("vacuum" OR "vacuum assisted" OR "vacuum-assisted" OR "ATEC*" OR "Brevera*" OR "Eviva*" OR "Celero*" OR "Mammotome*" OR "Elite*" OR "Revolve*" OR "EnCor*" OR "EnCor Enspire*" OR "EnCor Ultra*" OR "Vacora*" OR "EleVation*") AND ("breast biops*" OR "biops*" OR "core biops*" OR "excision" OR "device" OR "biopsy device" OR "suction biops*" OR "aspiration biops*")) OR "VAB" OR "VABB" OR "VAE" OR "VACNB" OR "VACB" OR "VALCB" OR "DVA") AND ((("core needle" OR "core-needle" OR "needle core" OR "core" OR "large core" OR "large-core" OR "percutaneous" OR "percutaneous core" OR "spring-loaded" OR "spring loaded" OR "spring-activated" OR "spring activated" OR "automated gun" OR "Sertera*" OR "EvoCore*" OR "Magnum*" OR "Mission*" OR "HistoCore*" OR "MaxCore*" OR "Max-Core*" OR "Monopty*" OR "Marquee*") AND ("biops*" OR "breast biops*")) OR "NCB" OR "CNB" OR "CB" OR "CCNB" OR "ACNB" OR "Biopsy, Large-Core Needle"[MeSH Terms] OR "core-biopsy" OR (("fine-needle" OR "fine needle") AND ("aspiration" OR "aspiration cytology" OR "aspiration biops*")) OR "Biopsy, Fine-Needle"[Mesh] OR "FNA" OR "BFNA" OR "FNAC" OR "FNAB" OR "Chiba*")) AND ("diagnostic management" OR "diagnostic performance" OR "diagnostic accuracy" OR "diagnostic yield" OR "accuracy" OR "sensitivity" OR "true positive rate" OR "true-positive rate" OR "TPR" OR "true positives" OR "true-positives" OR "sensitivity and specificity"[MeSH Terms] OR "sensitivity" OR "true negative rate" OR "true-negative rate" OR "TNR" OR "true negatives" OR "true-negatives" OR "false positive rate" OR "false-positive rate" OR "FPR" OR "false positives" OR "false-positives" OR "false negative rate" OR "false-negative rate" OR "FNR" OR "false negatives" OR "false-negatives" OR "positive predictive value" OR "PPV" OR "positive predictive" OR "positive test" OR "predictive value of tests"[MeSH Terms] OR "negative predictive value" OR "NPV" OR "negative predictive" OR "negative test" OR "area under curve"[MeSH Terms] OR "area under curves"[MeSH Terms] OR "area under the curve" OR "AUC" OR "receiver operat*" OR "underestimation rate*" OR "underestimate rate*" OR "cancer underestimation" OR "diagnostic underestimation" OR "diagnostic upgrade" OR "histologic upgrade" OR "upgrade rate*" OR "understaging" OR "upstaging" OR "disconcordance" OR "disconcordance rate*" OR "concordance" OR "concordance rate*" OR "agreement rate*" OR "underestimation" OR "upgrade" OR "upgrade risk" OR "rate of upgrade" OR "under-evaluation*" OR "under evaluation*" OR "missed cancer" OR (("ductal carcinoma in situ" OR "DCIS" OR "atypical ductal hyperplasia" OR "ADH") AND ("underestimation rate*" OR "underestimat*" OR "upgrade rate" OR "upgrade")) OR "repeat biopsy rate*" OR "biopsy failure rate*" OR "rebiopsy rate*" OR "repeat biops*" OR "calcification retrieval rate*" OR "calcification retrieval" OR "microcalcification retrieval rate*" OR "microcalcification retrieval" OR "retrieval failure" OR "success rate*" OR "complication*" OR "hematoma" OR "bleeding*" OR "infection*" OR "pain" OR "bruising" OR "workflow" OR "efficac*" OR "time under compression" OR "time" OR "minute*" OR ("time" AND "compression") OR "mortality" OR "died" OR "dead" OR "deceased" OR "morbidity" OR "quality of life" OR "quality of life"[MeSH Terms] OR "QoL" OR "QOL" OR "HRQOL" OR "HRQoL" OR "Quality-Adjusted Life Years"[Mesh] OR "Cost-Effectiveness Analysis"[Mesh] OR "Economic Factors"[Mesh] OR "cost-effectiveness*" OR "econom*" OR "Costs and Cost Analysis"[Mesh] OR "Cost-Benefit Analysis"[Mesh] OR "cost*") AND hasabstract[text] AND English[lang] AND ("1995/01/01"[PDAT] : "3000/12/31"[PDAT])

Search strategy – Cochrane Library

#1 (("vacuum" OR "vacuum assisted" OR "vacuum-assisted" OR "ATEC" OR "Brevera" OR "Eviva" OR "Celero" OR "Mammotome" OR "Elite" OR "Revolve" OR "EnCor" OR "EnCor Enspire" OR "EnCor Ultra" OR "Vacora" OR "EleVation") AND ("breast biopsy" OR "breast biopsies" OR "biopsy" OR "biopsies" OR "core biopsy" OR "core biopsies" OR "excision" OR "device" OR "biopsy device" OR "suction biopsy" OR "suction biopsies" OR "aspiration biopsy" OR "aspiration biopsies")) OR “VAB” OR “VABB” OR “VAE” OR “VACNB” OR “VACB” OR “VALCB” OR "DVA"

#2 MeSH descriptor: [Biopsy, Large-Core Needle] explode all trees

#3 MeSH descriptor: [Biopsy, Fine-Needle] explode all trees

#4 (("core needle" OR "core-needle" OR "needle core" OR "core" OR "large core" OR "large-core" OR "percutaneous" OR "percutaneous core" OR "spring-loaded" OR "spring loaded" OR "spring-activated" OR "spring activated" OR "automated gun" OR "Sertera" OR "EvoCore" OR "Magnum" OR "Mission" OR "HistoCore" OR "MaxCore" OR "Max-Core" OR "Monopty" OR "Marquee") AND ("biopsy" OR "biopsies" OR "breast biopsy" OR "breast biopsies")) OR "NCB" OR "CNB" OR "CB" OR "CCNB" OR "ACNB" OR "core-biopsy" OR (("fine-needle" OR "fine needle") AND ("aspiration" OR "aspiration cytology" OR "aspiration biopsy" OR "aspiration biopsies")) OR "FNA" OR "BFNA" OR "FNAC" OR "FNAB" OR "Chiba" OR #2 OR #3

#5 MeSH descriptor: [Sensitivity and Specificity] explode all trees

#6 MeSH descriptor: [Area Under Curve] explode all trees

#7 MeSH descriptor: [Predictive Value of Tests] explode all trees

#8 "diagnostic management" OR "diagnostic performance" OR "diagnostic accuracy" OR "diagnostic yield" OR "accuracy" OR "sensitivity" OR "true positive rate" OR "true-positive rate" OR "TPR" OR "true positives" OR "true-positives" OR "sensitivity" OR "true negative rate" OR "true-negative rate" OR "TNR" OR "true negatives" OR "true-negatives" OR "false positive rate" OR "false-positive rate" OR "FPR" OR "false positives" OR "false-positives" OR "false negative rate" OR "false-negative rate" OR "FNR" OR "false negatives" OR "false-negatives" OR "positive predictive value" OR "PPV" OR "positive predictive" OR "positive test" OR "negative predictive value" OR "NPV" OR "negative predictive" OR "negative test" OR "area under the curve" OR "AUC" OR "receiver operator" OR #5 OR #6 OR #7

#9 "underestimation rate" OR "underestimation rates" OR "underestimate rate" OR "underestimate rates" OR "cancer underestimation" OR "diagnostic underestimation" OR "diagnostic upgrade" OR "histologic upgrade" OR "upgrade rate" OR "upgrade rates" OR "understaging" OR "upstaging" OR "disconcordance" OR "disconcordance rate" OR "disconcordance rates" OR "concordance" OR "concordance rate" OR "concordance rates" OR "agreement rate" OR "agreement rates" OR "underestimation" OR "upgrade" OR "upgrade risk" OR "rate of upgrade" OR "under-evaluation" OR "under evaluation" OR "missed cancer" OR (("ductal carcinoma in situ" OR "DCIS" OR "atypical ductal hyperplasia" OR "ADH") AND ("underestimation rate" OR "underestimation rates" OR "underestimate" OR "underestimation" OR "upgrade rate" OR "upgrade"))

#10 "repeat biopsy rate" OR "repeat biopsy rates" OR "biopsy failure rate" OR "biopsy failure rates" OR "rebiopsy rate" OR "rebiopsy rates" OR "repeat biopsy" OR "repeat biopsies"

#11 "calcification retrieval rate" OR "calcification retrieval rates" OR "calcification retrieval" OR "microcalcification retrieval rate" OR "microcalcification retrieval rates" OR "microcalcification retrieval" OR "retrieval failure" OR "success rate" OR "success rates"

#12 "complication" OR "complications" OR "hematoma" OR "bleeding" OR "infection" OR "infections" OR "pain" OR "bruising"

#13 "workflow" OR "efficacy" OR "time under compression" OR "time" OR "minute" OR "minutes" OR ("time" AND "compression")

#14 MeSH descriptor: [Quality of Life] explode all trees

#15 MeSH descriptor: [Quality-Adjusted Life Years] explode all trees

#16 "mortality" OR "died" OR "dead" OR "deceased" OR "morbidity" OR "quality of life" OR "QoL" OR "QOL" OR "HRQOL" OR "HRQoL" OR #14 OR #15

#17 MeSH descriptor: [Cost-Effectiveness Analysis] explode all trees

#18 MeSH descriptor: [Economic Factors] explode all trees

#19 MeSH descriptor: [Costs and Cost Analysis] explode all trees

#20 MeSH descriptor: [Cost-Benefit Analysis] explode all trees

#21 "cost-effectiveness" OR "economic" OR "cost" OR "costs" OR #17 OR #18 OR #19 OR #20

#22 #8 OR #9 OR #10 OR #11 OR #12 OR #13 OR #16 OR #21

#23 #1 AND #4 AND #22

#24 #1 AND #4 AND #22 with Cochrane Library publication date Between Jan 1995 and Dec 2024

S2. PRISMA-DTA checklist

Section/topic	#	PRISMA-DTA Checklist Item	Reported on page #
**TITLE / ABSTRACT**			
Title	1	Identify the report as a systematic review (+/- meta-analysis) of diagnostic test accuracy (DTA) studies.	Title
Abstract	2	Abstract: See PRISMA-DTA for abstracts.	1
**INTRODUCTION**			
Rationale	3	Describe the rationale for the review in the context of what is already known.	3 - 4
Clinical role of index test	D1	State the scientific and clinical background, including the intended use and clinical role of the index test, and if applicable, the rationale for minimally acceptable test accuracy (or minimum difference in accuracy for comparative design).	3
Objectives	4	Provide an explicit statement of question(s) being addressed in terms of participants, index test(s), and target condition(s).	4
**METHODS**			
Protocol and registration	5	Indicate if a review protocol exists, if and where it can be accessed (e.g., Web address), and, if available, provide registration information including registration number.	5
Eligibility criteria	6	Specify study characteristics (participants, setting, index test(s), reference standard(s), target condition(s), and study design) and report characteristics (e.g., years considered, language, publication status) used as criteria for eligibility, giving rationale.	5
Information sources	7	Describe all information sources (e.g., databases with dates of coverage, contact with study authors to identify additional studies) in the search and date last searched.	5
Search	8	Present full search strategies for all electronic databases and other sources searched, including any limits used, such that they could be repeated.	ESM, S1
Study selection	9	State the process for selecting studies (i.e., screening, eligibility, included in systematic review, and, if applicable, included in the meta-analysis).	5
Data collection process	10	Describe method of data extraction from reports (e.g., piloted forms, independently, in duplicate) and any processes for obtaining and confirming data from investigators.	5-6
Definitions for data extraction	11	Provide definitions used in data extraction and classifications of target condition(s), index test(s), reference standard(s) and other characteristics (e.g. study design, clinical setting).	6
Risk of bias and applicability	12	Describe methods used for assessing risk of bias in individual studies and concerns regarding the applicability to the review question.	6
Diagnostic accuracy measures	13	State the principal diagnostic accuracy measure(s) reported (e.g. sensitivity, specificity) and state the unit of assessment (e.g. per-patient, per-lesion).	6
Synthesis of results	14	Describe methods of handling data, combining results of studies and describing variability between studies. This could include, but is not limited to: a) handling of multiple definitions of target condition. b) handling of multiple thresholds of test positivity, c) handling	6-8

		multiple index test readers, d) handling of indeterminate test results, e) grouping and comparing tests, f) handling of different reference standards	
Meta-analysis	D2	Report the statistical methods used for meta-analyses, if performed.	7
Additional analyses	16	Describe methods of additional analyses (e.g., sensitivity or subgroup analyses, meta-regression), if done, indicating which were pre-specified.	7
**RESULTS**			
Study selection	17	Provide numbers of studies screened, assessed for eligibility, included in the review (and included in meta-analysis, if applicable) with reasons for exclusions at each stage, ideally with a flow diagram.	9
Study characteristics	18	For each included study provide citations and present key characteristics including: a) participant characteristics (presentation, prior testing), b) clinical setting, c) study design, d) target condition definition, e) index test, f) reference standard, g) sample size, h) funding sources	9
Risk of bias and applicability	19	Present evaluation of risk of bias and concerns regarding applicability for each study.	9
Results of individual studies	20	For each analysis in each study (e.g. unique combination of index test, reference standard, and positivity threshold) report 2x2 data (TP, FP, FN, TN) with estimates of diagnostic accuracy and confidence intervals, ideally with a forest or receiver operator characteristic (ROC) plot.	9-12
Synthesis of results	21	Describe test accuracy, including variability; if meta-analysis was done, include results and confidence intervals.	9-12
Additional analysis	23	Give results of additional analyses, if done (e.g., sensitivity or subgroup analyses, meta-regression; analysis of index test: failure rates, proportion of inconclusive results, adverse events).	9-12
**DISCUSSION**			
Summary of evidence	24	Summarize the main findings including the strength of evidence.	13
Limitations	25	Discuss limitations from included studies (e.g. risk of bias and concerns regarding applicability) and from the review process (e.g. incomplete retrieval of identified research).	14-15
Conclusions	26	Provide a general interpretation of the results in the context of other evidence. Discuss implications for future research and clinical practice (e.g. the intended use and clinical role of the index test).	15-16
**FUNDING**			
Funding	27	For the systematic review, describe the sources of funding and other support and the role of the funders.	Discloure paragraph

Adapted From: McInnes MDF, Moher D, Thombs BD, McGrath TA, Bossuyt PM, The PRISMA-DTA Group (2018). Preferred Reporting Items for a Systematic Review and Meta-analysis of Diagnostic Test Accuracy Studies: The PRISMA-DTA Statement. JAMA. 2018 Jan 23;319(4):388-396. doi: 10.1001/jama.2017.19163.

S3. Extraction sheet according to checklist of the data extraction for complex meta-analysis (DECiMAL)

This supplemental material S3 is provided as a separate MS Excel file.

S4: Results of the sensitivity analyses for comparing any imaging-guided VABB to any imaging-guided CNB

All studies included / Removing each study	N	Pooled Risk Ratio	Random 95% CI	p-value	favor
**ADH underestimation rate (VABB vs CNB)**					
All studies included	22	0.63	0.55, 0.72	<0.01	VABB
Badan 2016	21	0.63	0.55, 0.72	<0.01	VABB
Bae 2015	21	0.63	0.55, 0.72	<0.01	VABB
Berg 2001	21	0.64	0.56, 0.73	<0.01	VABB
Bertani 2020	21	0.63	0.55, 0.71	<0.01	VABB
Burbank 1997	21	0.63	0.55, 0.72	<0.01	VABB
Darling 2000	21	0.64	0.56, 0.73	<0.01	VABB
Huang 2011	21	0.63	0.55, 0.72	<0.01	VABB
Jackman 1997	21	0.65	0.57, 0.74	<0.01	VABB
Kil 2008	21	0.63	0.55, 0.72	<0.01	VABB
Ko 2008	21	0.62	0.55, 0.71	<0.01	VABB
Liberman 2001	21	0.63	0.55, 0.72	<0.01	VABB
Londero 2011	21	0.63	0.55, 0.72	<0.01	VABB
Oktay 2023	21	0.62	0.54, 0.72	<0.01	VABB
Park 2022	21	0.65	0.57, 0.64	<0.01	VABB
Rageth 2019	21	0.64	0.55, 0.74	<0.01	VABB
Seely 2017	21	0.63	0.55, 0.72	<0.01	VABB
Seo 2017	21	0.63	0.55, 0.72	<0.01	VABB
Tothova 2013	21	0.63	0.55, 0.72	<0.01	VABB
Willers 2023	21	0.62	0.54, 0.71	<0.01	VABB
Zannis 1998	21	0.63	0.55, 0.72	<0.01	VABB
Zhang 2023	21	0.57	0.48, 0.67	<0.01	VABB
Zhao 2003	21	0.63	0.55, 0.72	<0.01	VABB

All studies included / Removing each study	N	Pooled Risk Ratio	Random 95% CI	p-value	favor
**DCIS underestimation rate (VABB vs CNB)**					
All studies included	27	0.47	0.39, 0.58	<0.01	VABB
Badan 2016	26	0.47	0.38, 0.58	<0.01	VABB
Bae 2015	26	0.48	0.39, 0.60	<0.01	VABB
Bundred 2016	26	0.47	0.38, 0.58	<0.01	VABB
Burbank 1997	26	0.48	0.39, 0.59	<0.01	VABB
Darling 2000	26	0.47	0.38, 0.58	<0.01	VABB
Dória 2018	26	0.45	0.37, 0.56	<0.01	VABB
Elsharkawy 2020	26	0.48	0.39, 0.59	<0.01	VABB
Hsieh 2023	26	0.50	0.41, 0.60	<0.01	VABB
Huang 2011	26	0.48	0.39, 0.59	<0.01	VABB
Jackman 2001	26	0.46	0.37, 0.58	<0.01	VABB
Kim 2012	26	0.46	0.37, 0.58	<0.01	VABB
Lee 2013	26	0.51	0.42, 0.61	<0.01	VABB
Liberman 2001	26	0.46	0.37, 0.57	<0.01	VABB
Mannu 2019	26	0.46	0.37, 0.57	<0.01	VABB
Marques 2019	26	0.48	0.39, 0.59	<0.01	VABB
Seely 2017	26	0.48	0.39, 0.59	<0.01	VABB
Seo 2017	26	0.48	0.39, 0.59	<0.01	VABB
Sheng 2020	26	0.46	0.37, 0.56	<0.01	VABB
Sim 2015	26	0.45	0.37, 0.56	<0.01	VABB
Suh 2012	26	0.48	0.39, 0.60	<0.01	VABB
Szynglarewicz 2015	26	0.47	0.38, 0.58	<0.01	VABB
Tothova 2013	26	0.48	0.39, 0.59	<0.01	VABB
Won 1999	26	0.47	0.38, 0.58	<0.01	VABB
Yashima 2023	26	0.46	0.37, 0.57	<0.01	VABB
Zannis 1998	26	0.48	0.39, 0.59	<0.01	VABB
Zhang 2023	26	0.48	0.38, 0.59	<0.01	VABB
Zou 2019	26	0.50	0.41, 0.60	<0.01	VABB

All studies included / Removing each study	N	Pooled Risk Ratio	Random 95% CI	p-value	favor
**Repeat biopsy rate (VABB vs CNB)**					
All studies included	10	0.78	0.69, 0.88	<0.01	VABB
Bundred 2016	9	0.77	0.71, 0.83	<0.01	VABB
Cho 2005	9	0.80	0.69, 0.93	<0.01	VABB
**Grady 2017**	**9**	**0.83**	**0.66, 1.05**	**0.13**	**VABB**
Liberman 2000	9	0.79	0.67, 0.92	<0.01	VABB
Philpotts 1999	9	0.79	0.71, 0.87	<0.01	VABB
Philpotts 2003	9	0.76	0.71, 0.82	<0.01	VABB
Poole 2015	9	0.77	0.69, 0.86	<0.01	VABB
Velanovich V 1999	9	0.78	0.67, 0.90	<0.01	VABB
Yashima 2023	9	0.79	0.69, 0.91	<0.01	VABB

All studies included / Removing each study	N	Pooled Risk Ratio	Random 95% CI	p-value	favor
**Concordance rate (VABB vs CNB)**					
All studies included	12	1.07	1.04, 1.11	<0.01	VABB
Brenner 2000	11	1.08	1.04, 1.12	<0.01	VABB
Bundred 2016	11	1.08	1.04, 1.12	<0.01	VABB
Elsharkawy 2020	11	1.08	1.04, 1.12	<0.01	VABB
Huang 2011	11	1.07	1.03, 1.11	<0.01	VABB
Middleton 2003	11	1.07	1.04, 1.11	<0.01	VABB
Povoski 2011	11	1.05	1.03, 1.08	<0.01	VABB
Seely 2017	11	1.07	1.03, 1.11	<0.01	VABB
Soo 1999	11	1.07	1.04, 1.11	<0.01	VABB
Won 1999	11	1.07	1.03, 1.11	<0.01	VABB
Zannis 1998	11	1.07	1.03, 1.11	<0.01	VABB
Zhang 2023	11	1.08	1.04, 1.13	<0.01	VABB
Zou 2019	11	1.08	1.03, 1.13	<0.01	VABB

All studies included / Removing each study	N	Pooled Risk Ratio	Random 95% CI	p-value	favor
**Calcification retrieval rate (VABB vs CNB)**					
All studies included	9	1.11	1.06, 1.16	<0.01	VABB
Bae 2015	8	1.11	1.06, 1.17	<0.01	VABB
Berg 2001	8	1.12	1.07, 1.17	<0.01	VABB
Bundred 2016	8	1.12	1.06, 1.17	<0.01	VABB
Huang 2011	8	1.09	1.05, 1.13	<0.01	VABB
Jackman 2006	8	1.10	1.05, 1.15	<0.01	VABB
Liberman L 2001	8	1.12	1.06, 1.17	<0.01	VABB
Meyer JE 1997	8	1.11	1.05, 1.17	<0.01	VABB
Philpotts 1999	8	1.12	1.06, 1.17	<0.01	VABB
Reynolds 1998	8	1.10	1.06, 1.16	<0.01	VABB

All studies included / Removing each study	N	Pooled Risk Ratio	Random 95% CI	p-value	Favor
**False-negative rate (VABB vs CNB)**					
All studies included	10	0.67	0.43, 1.04	0.07	VABB
Becker 2006	9	0.64	0.39, 1.05	0.08	VABB
Ciatto 2007	9	0.72	0.43, 1.22	0.23	VABB
Fuentes 2019	9	0.66	0.41, 1.05	0.08	VABB
Huang 2011	9	0.70	0.44, 1.11	0.13	VABB
Lacambra 2012	9	0.66	0.41, 1.06	0.08	VABB
**La Forgia 2020**	**9**	**0.57**	**0.41, 0.79**	**<0.01**	**VABB**
Shin 2008	9	0.69	0.44, 1.09	0.11	VABB
Tian 2024	9	0.71	0.46, 1.09	0.12	VABB
Tothova 2013	9	0.69	0.43, 1.11	0.13	VABB
Zhang 2023	9	0.66	0.36, 1.21	0.18	VABB

S5: Results comparing X-ray guided VABB to any imaging-guided CNB

(a) Summary of results comparing X-ray *guided VABB to any imaging-guided CNB: ADH underestimation rate, DCIS underestimation rate, repeat biopsy rate, concordance rate, calcification retrieval rate, and false negative rate*

	No. studies	*Risk ratio (95% CI) using REM, p-value*	*favor*
ADH underestimation rate	15	0.53 (0.42-0.66), p<.01	VABB
DCIS underestimation rate	13	0.42 (0.30-0.60), p<.01	VABB
Repeat biopsy rate	3	0.94 (0.68-1.29), p=.69	-
Concordance rate	7	1.08 (0.99-1.17), p=.07	-
Calcification retrieval rate	9	1.09 (1.02-1.15), p<.01	VABB
False negative rate	2	0.30 (0.08-1.17), p=.08	-

(b) Summary of results comparing X-ray *guided VABB to any imaging-guided CNB: sensitivity and specificity*

	No. studies	*Pooled values (95% CI) using REM*
Sensitivity	2	xVABB: 0.91 (0.80-1.00)<br>xCNB: 0.68 (0.56-0.80)
Specificity	2	xVABB: 1.00 (0.97-1.00)<br>xCNB: 1.00 (0.99-1.00)

ADH*: atypical ductal hyperplasia;* ***DCIS****: ductal carcinoma in-situ;* ***REM****: random effects model;* ***VABB****: vacuum-assisted breast biopsy;* ***CNB****: core needle biopsy;* ***xVABB****:* X-ray *guided VABB,* ***xCNB****:* X-ray *guided CNB*

S6: Detailed study-level QUADAS-2 assessment reporting individual domain-level risk-of-bias and applicability judgments for each included study

This supplemental material S6 is provided as a separate MS Excel file.

S7. Funnel plots

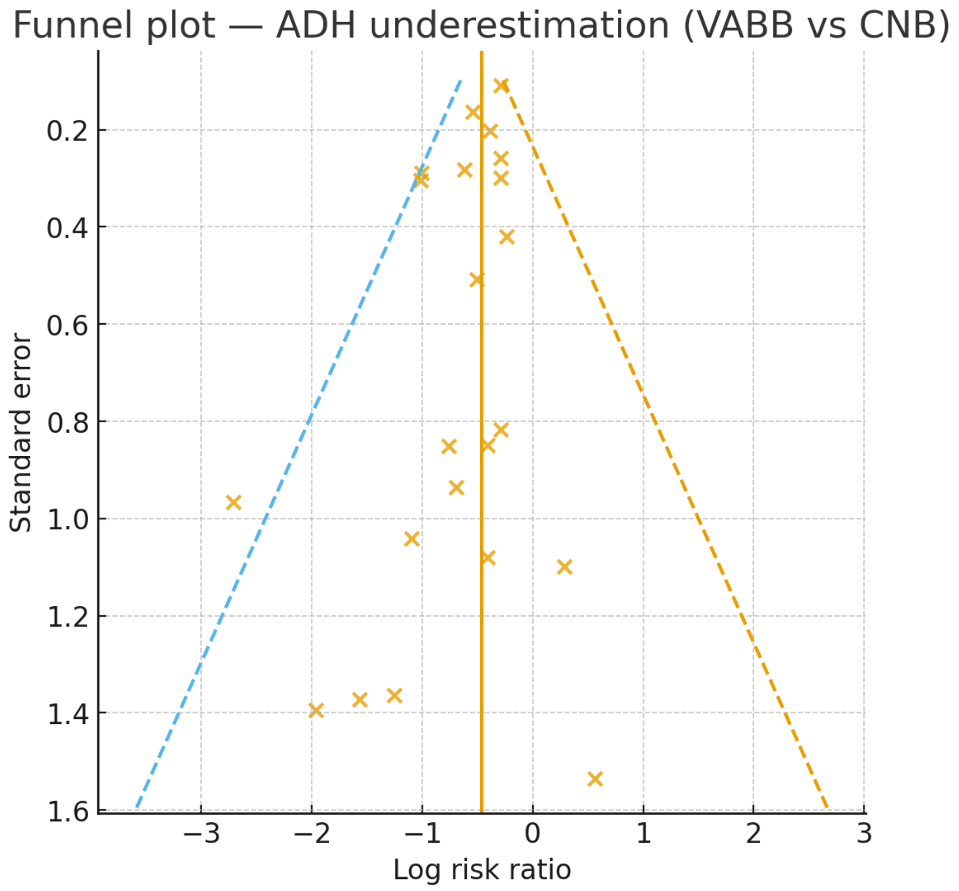

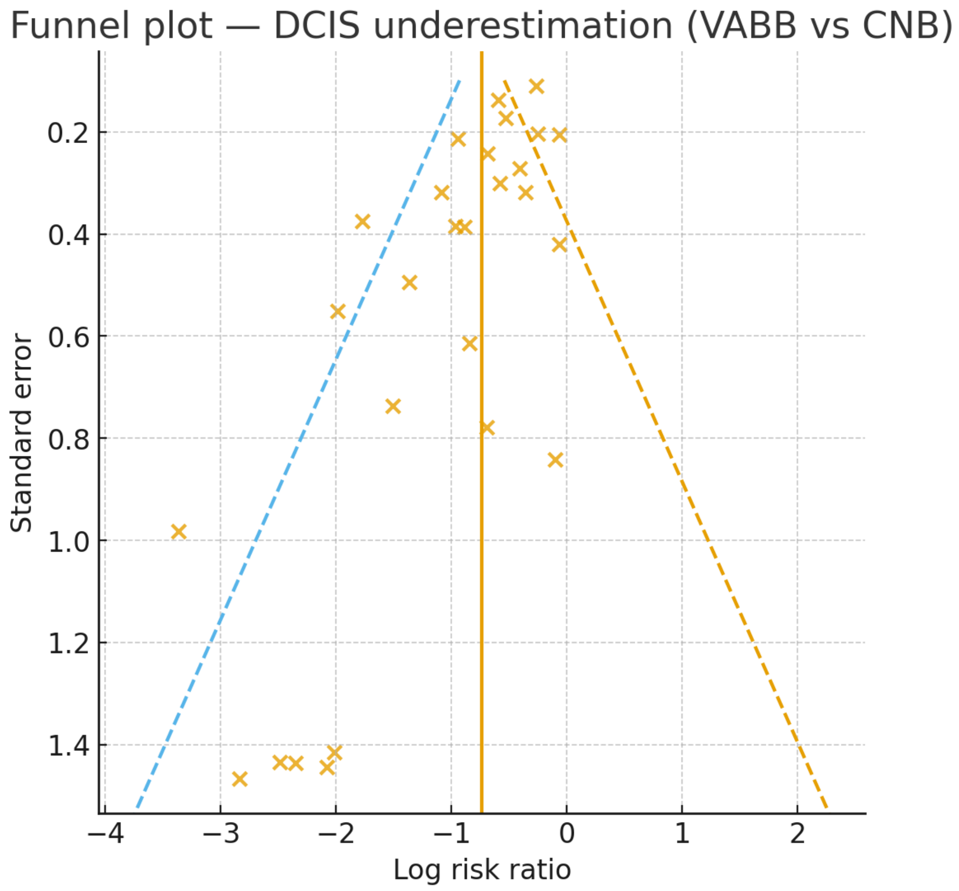

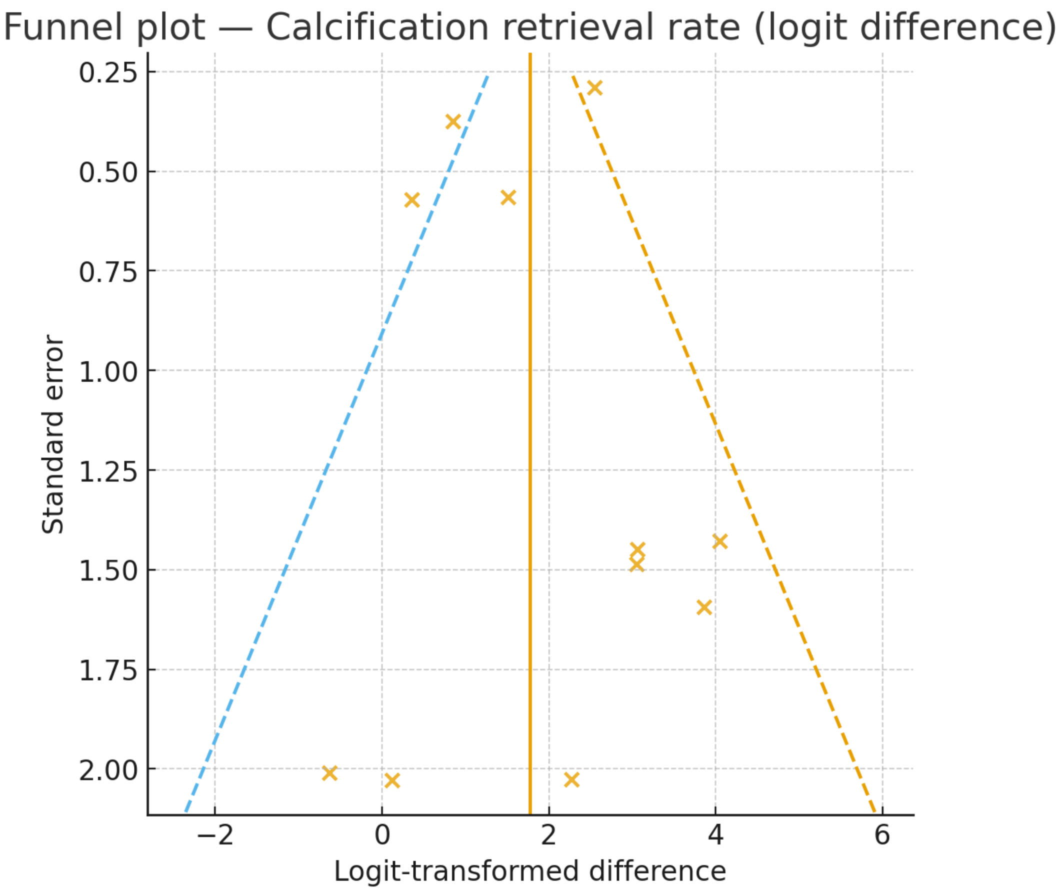
Funnel plot — Calcification retrieval rate (logit difference)
Standard error
0.25
0.50
0.75
1.00
1.25
1.50
1.75
2.00
−2
0
2
4
6
Logit-transformed difference

+

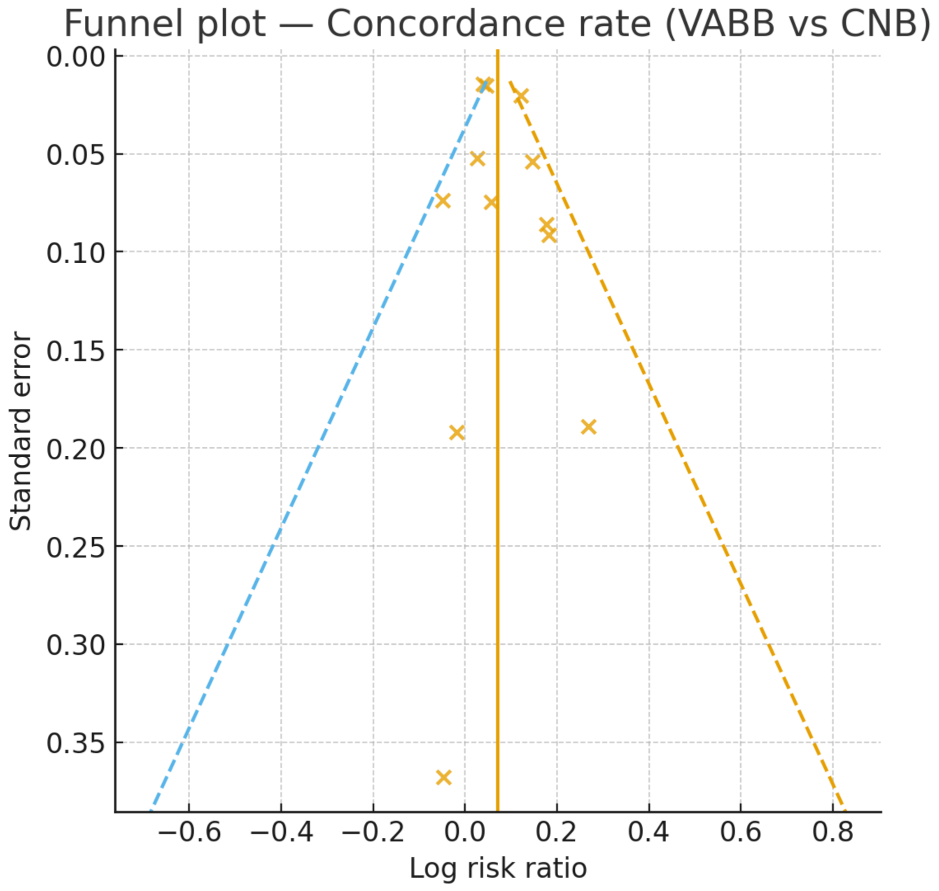
Funnel plot — Concordance rate (VABB vs CNB)
Standard error
0.00
0.05
0.10
0.15
0.20
0.25
0.30
0.35
−0.6
−0.4
−0.2
0.0
0.2
0.4
0.6
0.8
Log risk ratio

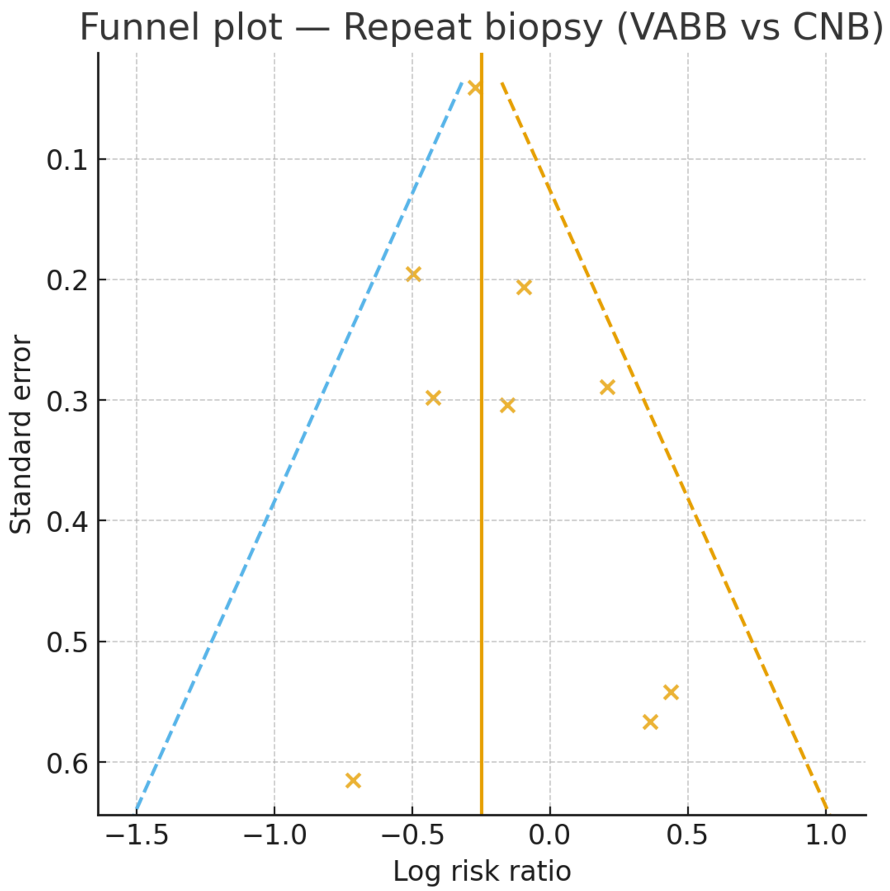
Funnel plot — Repeat biopsy (VABB vs CNB)
Standard error
0.1
0.2
0.3
0.4
0.5
0.6
−1.5
−1.0
−0.5
0.0
0.5
1.0
Log risk ratio
